# Repression of *Acinetobacter baumannii* DNA damage response requires DdrR-assisted binding of UmuDAb dimers to atypical SOS box

**DOI:** 10.1128/jb.00432-23

**Published:** 2024-05-10

**Authors:** Belinda Candra, Deborah Cook, Janelle Hare

**Affiliations:** 1 Baylor College of Medicine, Houston, Texas, USA; 2 Department of Biology and Chemistry, Morehead State University, Morehead, Kentucky, USA; University of Southern California, Los Angeles, California, USA

**Keywords:** DNA damage response, LexA, UmuDAb, DdrR, *Acinetobacter baumannii*, gene repression

## Abstract

The DNA damage response of the multi-drug-resistant nosocomial pathogen *Acinetobacter baumannii* possesses multiple features that distinguish it from the commonly used LexA repression system. These include the absence of LexA in this genus, the evolution of a UmuD polymerase manager into the UmuDAb repressor of error-prone polymerases, the use of a corepressor unique to *Acinetobacter* (DdrR), and an unusually large UmuDAb binding site. We defined cis- and trans-acting factors required for UmuDAb DNA binding and gene repression, and tested whether DdrR directly enhances its DNA binding. We used DNA binding assays to characterize UmuDAb’s binding to its proposed operator present upstream of the six co-repressed *umuDC* or *umuC* genes. UmuDAb bound tightly and cooperatively to this site with ~10-fold less affinity than LexA. DdrR enhanced the binding of both native and dimerization-deficient UmuDAb forms, but only in greater than equimolar ratios relative to UmuDAb. UmuDAb mutants unable to dimerize or effect gene repression showed impaired DNA binding, and a strain expressing the G124D dimerization mutant could not repress transcription of the UmuDAb-DdrR regulon. Competition electrophoretic mobility shift assays conducted with mutated operator probes showed that, unlike typical SOS boxes, the UmuDAb operator possessed a five-base pair central core whose sequence was more crucial for binding than the flanking palindrome. The presence of only one of the two flanking arms of the palindrome was necessary for UmuDAb binding. Overall, the data supported a model of an operator with two UmuDAb binding sites. The distinct characteristics of UmuDAb and its regulated promoters differ from the typical LexA repression model, demonstrating a novel method of repression.

**IMPORTANCE:**

*Acinetobacter baumannii* is a gram-negative bacterium responsible for hospital-acquired infections. Its unique DNA damage response can activate multiple error-prone polymerase genes, allowing it to gain mutations that can increase its virulence and antibiotic resistance. The emergence of infectious strains carrying multiple antibiotic resistance genes, including carbapenem resistance, lends urgency to discovering and developing ways to combat infections resistant to treatment with known antibiotics. Deciphering how the regulators UmuDAb and DdrR repress the error-prone polymerases could lead to developing complementary treatments to halt this mechanism of generating resistance.

## INTRODUCTION

**Fig 1:**
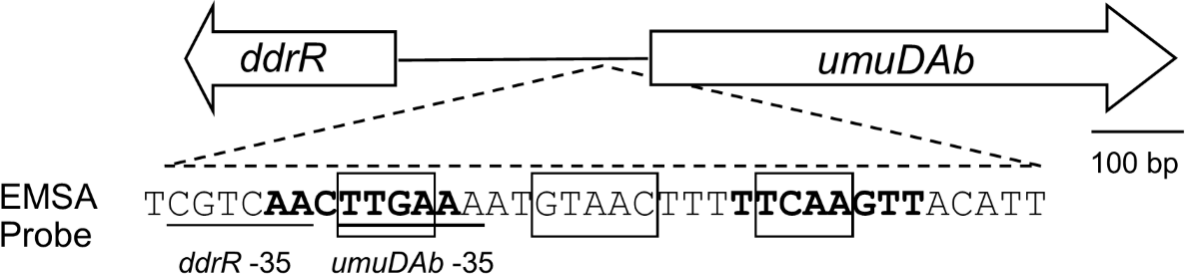
The shared *umuDAb* and *ddrR* promoter region of *A. baumannii* ATCC 17978, showing the sequence and location of the electrophoretic mobility shift assay (EMSA) probe. The 37 bp oligonucleotide probe for mobility shift assays is shown, with boxes around the consensus elements identified in transcriptome studies (5). Inverted repeats corresponding to those observed in *A. baylyi* (6) are depicted in bold; the –35 of *ddrR* (on the opposite strand) and *umuDAb* (TTGAAA) are underlined (2).

After DNA damage, the multi-drug-resistant pathogen *Acinetobacter baumannii* activates an SOS response, inducing ~150 genes, including multiple error-prone polymerases that conduct SOS mutagenesis. Like a minority of bacteria, however, the genus *Acinetobacter* does not encode a LexA protein that controls the repression of this entire regulon (1). Instead, to control and repress these polymerases, *A. baumannii* employs the combined actions of a repressor protein, UmuDAb, and a small protein, DdrR, which is unique to the *Acinetobacter* genus. DdrR enhances the UmuDAb-mediated repression of target *umuDC* operons (*A1S_0636–0637*, *A1S_1173–1174*) and *umuC* genes (*A1S_2008*, *A1S_2015*) as well as the divergently transcribed *ddrR* and *umuDAb* regulators themselves (2) (Fig. 1). DdrR does not bind directly to UmuDAb-DdrR regulated promoters but does bind to UmuDAb (3), although the mechanism of UmuDAb corepression with DdrR has not been determined. A precedent for a corepressor aiding LexA binding has been seen in the small bacteriophage-encoded protein gp7, which binds to LexA in *Bacillus thuringiensis* as a dimer of dimers and modulates LexA DNA binding (4).

Multiple features of the *Acinetobacter* UmuDAb-DdrR regulation of DNA damage-responsive genes exist that distinguish it from the LexA SOS response system. It appears that in *A. baylyi* ADP1, *umuDAb* evolved from a *umuDC* operon that became mutated by transposition and possibly by acquisition of a DNA binding N-terminal domain (NTD) (6). The regulatory action of UmuDAb was initially overlooked because of this annotation of *umuDAb* as *umuD* in conserved domain searches, and because BLASTp algorithms do not predict any DNA binding motif such as a helix-turn-helix (HTH) motif in UmuDAb. However, AlphaFold and I-TASSER protein modeling servers predict a linear and three-dimensional arrangement of helices in the UmuDAb NTD that resemble their prediction for the LexA HTH motif required for DNA binding (7). Mutations disrupting either of these putative helices derepress gene expression, with modification of helix 2 resulting in higher uninduced expression of the UmuDAb-DdrR regulon than helix 1 (8). This showed that these NTD helices are required for UmuDAb to regulate DNA damage-inducible genes (9). However, their role in UmuDAb binding to DNA has yet to be tested experimentally.

Although the mechanism of UmuDAb binding DNA is not understood, several structural determinants facilitate actions similar to those of the Lex repressor, such as its dimerization (10) and intramolecular self-cleavage mediated by activated RecA after DNA damage (11). For example, UmuDAb forms dimers through interactions of the C-terminal ~12 amino acids of its C-terminal domain (CTD), but also relies on Gly-124 (10), which is located at an equivalent location in the protein as the LexA dimer interface (12). These two hybrid studies indicate that a free C-terminus of UmuDAb is more important in UmuDAb dimer formation than G124, which, in turn, is more important than Asn-100, which allows dimerization of the UmuD component of the error-prone polymerase (13).

In addition to the unusual UmuDAb NTD structure, the UmuDAb binding site possesses a unique size and structure. In many gammaproteobacterial species, the consensus operator sequence (SOS box) to which the LexA repressor binds consists of a four-base pair palindrome separated by an eight-base pair spacer (e.g., CTGT(N_8_)ACAG) (14). However, the putative UmuDAb operator, which can be termed an SOS box, given its repression of DNA damage-responsive genes in the absence of DNA damage (9), is larger and more complex than the *Escherichia coli* SOS box. Early studies observed that *Acinetobacter baylyi* strain ADP1 possesses not four, but eight-bp inverted repeats in the *ddrR-umuDAb* promoter region to which an unknown repressor might bind (6), as the genus *Acinetobacter* lacks a LexA protein (15). In *A. baumannii*, these inverted repeats are required for target gene repression by UmuDAb (9).

Electrophoretic mobility shift assays (EMSAs) using a 100 bp probe surrounding these inverted repeats identified a UmuDAb binding site in *A. baumannii* located upstream of all UmuDAb-DdrR repressed genes: TTGA(N_4_)GTWAC(N_4_)TCAA (5). This putative operator has an additional, conserved five base pairs at its core (5). Both a four-bp part of the inverted repeats we observed (6) (termed the “left” and “right” arms) and the five-bp GTWAC core were important for UmuDAb binding (5). However, these studies were not quantitative and could not determine the relative contributions of these elements to the *umuDAb-ddrR* operator.

Although we know that DdrR-UmuDAb interactions repress DNA damage-induced genes, many aspects of their coregulation have not been determined. We do not know whether the NTD helices necessary for gene repression and CTD that facilitate homodimer formation are required for UmuDAb to bind its atypically large operator, nor whether DdrR contributes to repression by aiding UmuDAb binding. To clarify the mechanisms of UmuDAb and DdrR repression, we used EMSA experiments to identify the relative contributions of the UmuDAb HTH and dimerization motifs in DNA binding, determine whether UmuDAb binding is affected by the presence of DdrR, and whether the inverted repeats and the core region were equally required for UmuDAb binding. We found that dimerization was more important for binding than the HTH motif, and that the core of the UmuDAb operator was more important than either of the individual inverted repeats flanking it. Furthermore, we observed DdrR to increase the binding of UmuDAb to its operator when present in greater than equimolar concentrations relative to UmuDAb.

## MATERIALS AND METHODS

### Protein production and purification

Recombinant proteins were produced from *umuDAb* (A1S_1389) and *ddrR* (A1S_1388) of *Acinetobacter baumannii* ATCC 17978 JH, our laboratory’s *A. baumannii* strain ATCC 17978 strain. The PCR-confirmed presence of the AbaAL44 accessory locus of 17978 in 17978 JH indicates that it closely matches 17978 UN (16). *umuDAb* and *ddrR* were PCR-amplified from 17978 JH genomic DNA (primers listed in Table 1) and cloned into the multi-cloning site of the expression vector pET-15b (Novagen) on *Xho*I (or *Nde*I) and *Bam*HI ends to yield pET15bUDAb and pET15DR. Recombinant proteins were overexpressed in *E. coli* strain BL21(DE3) RIL (New England Biolabs). Site-directed mutations of *umuDAb* in pET15bUDAb were constructed with the Agilent QuikChange Lightning Site-Directed Mutagenesis kit using mutagenic primers described previously for the HTH1 (*E24K*) and HTH2 (*K40P N41P*) mutations (9), the *N100D*, *G124D*, *W192X*, and *R201X* dimerization mutations (10), and the uncleavable mutant A83Y (8).

**TABLE 1:** Primers used in this study

Primer name	Sequence^*a*^	Purpose
UmuDAbXhoI	ATCGCTCGAGATGCCAAAGAAGAAAGAA	Cloning 17978 *umuDAb* into pET-15b
UmuDAbBamHI	AGGGATCCTTATCTCATTCGTTTGAG	Cloning 17978 *umuDAb* into pET-15b
ddrRNde	ATATCGCTCATATGAAAAATCAACGTGATGCAG	Cloning 17978 *ddrR* into pET-15b
ddrRstopBam	TATATAGGATCCAATTATGAGTGGGTAAGGGGATG	Cloning 17978 *ddrR* into pET-15b
MSAUDAbF	5-IRDye700-TCGTCAACTTGAAAATGTAACTTTTTCAAGTTACATT	Labeled wild-type UmuDAb binding site; EMSA
MSAUDAbR	5-IRDye700-AATGTAACTTGAAAAAGTTACATTTTCAAGTTGACGA	Labeled wild-type UmuDAb binding site; EMSA
UnlabelMSAUDAbF	TCAACTTGAAAATGTAACTTTTTCAAGTTAC	Wild-type unlabeled competitor UmuDAb binding site; EMSA
UnlabelMSAUDAbR	GTAACTTGAAAAAGTTACATTTTCAAGTTGA	Wild-type unlabeled competitor UmuDAb binding site; EMSA
MSA L1	AATGTAACTCAAAAAAGTTACATTTTCAAGTTGACGA	Unlabeled competitor mutated in the left arm of the UmuDAb binding site; EMSA
MSA L1R	TCGTCAACTTGAAAATGTAACTTTTTTGAGTTACATT	Unlabeled competitor mutated in the left arm of tthe UmuDAb binding site; EMSA
MSA L2	TCGTCAACCCAGAAATGTAACTTTTTCAAGTTACATT	Unlabeled competitor mutated in the left arm of the UmuDAb binding site; EMSA
MSA L2R	AATGTAACTTGAAAAAGTTACATTTCTGGGTTGACGA	Unlabeled competitor mutated in the left arm of the UmuDAb binding site; EMSA
MSA R1	AATGTAACTTGAAAAAGTTACATTTTTGAGTTGACGA	Unlabeled competitor mutated in the right arm of the UmuDAb binding site; EMSA
MSA R1R	TCGTCAACTCAAAAATGTAACTTTTTCAAGTTACATT	Unlabeled competitor mutated in the right arm of the UmuDAb binding site; EMSA
MSA R2	TCGTCAACTTGAAAATGTAACTTTTCTGGGTTACATT	Unlabeled competitor mutated in the right arm of the UmuDAb binding site; EMSA
MSA R2R	AATGTAACCCAGAAAAGTTACATTTTCAAGTTGACGA	Unlabeled competitor mutated in the right arm of the UmuDAb binding site; EMSA
MSA LR3	TCGTCAACCCAGAAATGTAACTTTTCTGGGTTACATT	Unlabeled competitor mutated in the left and right arms of UmuDAb binding site; EMSA
MSA LR3R	AATGTAACCCAGAAAAGTTACATTTCTGGGTTGACGA	Unlabeled competitor mutated in the left and right arms of UmuDAb binding site; EMSA
MSA C1	TCGTCAACTTGAAAATGCAGCTTTTTCAAGTTACATT	Unlabeled competitor mutated in the core of UmuDAb binding site; EMSA
MSA C1R	AATGTAACTTGAAAAAGCTGCATTTTCAAGTTGACGA	Unlabeled competitor mutated in the core of UmuDAb binding site; EMSA
MSA C2	TCGTCAACTTGAAAATTTAAATTTTTCAAGTTACATT	Unlabeled competitor mutated in the core of UmuDAb binding site; EMSA
MSA C2R	AATGTAACTTGAAAAATTTAAATTTTCAAGTTGACGA	Unlabeled competitor mutated in the core of UmuDAb binding site; EMSA
MSA C3	TCGTCAACTTGAAAATTCAGATTTTTCAAGTTACATT	Unlabeled competitor mutated in the core of UmuDAb binding site; EMSA
MSA C3R	AATGTAACTTGAAAAATCTGAATTTTCAAGTTGACGA	Unlabeled competitor mutated in the core of UmuDAb binding site; EMSA
L2C31-2 F	TCGTCAACCCAGAAATTCAACTTTTTCAAGTTACATT	Unlabeled competitor mutated in the left arm and core bp 1–2 of UmuDAb binding site; EMSA
L2C31-2 R	AATGTAACTTGAAAAAGTTGAATTTCTGGGTTGACGA	Unlabeled competitor mutated in the left arm and core bp 1–2 of UmuDAb binding site; EMSA
L2C34-5 F	TCGTCAACCCAGAAATGTAGATTTTTCAAGTTACATT	Unlabeled competitor mutated in the left arm and core bp 4–5 of UmuDAb binding site; EMSA
L2C34-5 R	AATGTAACTTGAAAAAATTACATTTCTGGGTTGACGA	Unlabeled competitor mutated in the left arm and core bp 4–5 of UmuDAb binding site; EMSA

^*a*^ Underlined sequences are mutated from wild-type sequences.

Cells bearing pET-15b plasmids in a native or mutated form were grown aerobically at 37°C in 50 mL lysogeny broth, supplemented with 30 µg/mL chloramphenicol and 100 µg/mL carbenicillin to an OD_600_ of 0.6. Protein synthesis was then induced with 1 mM isopropyl β-D-1-thiogalactopyranoside (IPTG). For UmuDAb expression, cultures were induced for 4 hours at 37°C. Cell cultures expressing DdrR were cooled to 20°C before IPTG induction and were grown for 18 hours at 20°C for a higher yield of soluble protein (3). Cell pellets were stored at −20°C overnight. Each pellet was thawed on ice, resuspended in 2 mL equilibration buffer (50 mM NaH_2_PO_4_ 300 mM NaCl), and lysed by sonication with a Fisher sonic dismembrator FB-120 while on ice using 10-second pulses at 10-second intervals at 60% for 2 minutes.

The proteins were affinity purified by loading 1 mL lysate onto 1 mL bed volume of pre-equilibrated TALON resin in TALON 2 mL columns. Proteins were washed and eluted using equilibration buffer and imidazole (10 mM for column equilibration, 20 mM for washes, and 250 mM for elution). Eluted proteins were then filter-dialyzed in Amicon Ultra-0.5 3K centrifugal filter devices in four 30-minute spins at 4°C adding 500 µL dialysis buffer (10 mM NaH_2_PO_4_ 300 mM NaCl, 1.8 mM KH_2_PO_4_, 2.7 mM KCl, and 2 mM dTT) after each spin until the last spin before recovery to <1 mM imidazole concentration. Proteins were then recovered with a 2-minute spin at 1,000 × *g* of the inverted filter column in a collection tube, quantified with the Qubit Protein Assay Kit (ThermoFisher Scientific), and stored at −80°C.

### EMSA assays

Fluorescently labeled probes comprising the UmuDAb binding site of *A. baumannii* ATCC 17978 were obtained from IDT, Inc. Each pair of complementary 5′ IRDye 700 end-labeled, HPLC-purified primers (Table 1) was individually diluted in IDTE 1XTe Solution (pH 7.5) to 100 pmol/µL, combined, and denatured in a heat block at 95°C in the dark for 5 minutes. The heat block was turned off and allowed to cool until reaching room temperature, after which the probe was diluted at 1:100 (for 5′ IRDye-labeled probes only), and aliquots were stored at −20°C (17). The final amount of annealed oligo was 200 fmol per 20 μL binding reaction.

Biorad 5% Polyacrylamide Mini-Protean TBE gels were pre-run for 90 minutes at 70 V in 0.5 × TBE buffer. EMSAs were performed with the Odyssey Infrared EMSA Kit (LI-COR). Binding reactions were performed in 10 × Binding Buffer (100 mM Tris, 500 mM KCl, 10 mM DTT, pH 7.5), 25 mM DTT, and 1 µg/µL Poly (dl·dC) from the Odyssey Infrared EMSA Kit (Li-COR) for 20 minutes at room temperature. [Where used, competitor oligonucleotides were used at 200× (i.e., undiluted after annealing) that of the labeled probe and added before the labeled probe.] Serial dilutions of varying concentrations of wild-type UmuDAb or the mutant UmuDAb HTH1, HTH2, N100D, G124D, W192X, or R201X proteins were added in 1 µL to the binding reaction. Bovine serum albumin (BSA) was obtained from New England Biolabs. 2 µL of Odyssey Orange 10× Loading Dye (Li-COR) was added to each reaction tube before the sample was loaded into the pre-run gel and run for 90 minutes at 70 V at 4°C. DNA-protein complexes were detected using a LI-COR Odyssey Fc Imager, and the fluorescent signal was analyzed with LI-COR ImageStudio 5.2 software to quantify binding. Experimental data were fit in GraphPad Prism 10.0.2 using non-linear regression with specific binding using the Hill slope and least squares fit. This method was chosen after the Prism “Compare models” function evaluated this model to be more effective than one-site-specific binding.

### RT-qPCR

RT-qPCR analyses were performed as previously described (15). Biological triplicates of *A. baumannii* 17978 JH strains (see Table 2) were grown in 3 mL overnight cultures at 37°C at 250 rpm in minimal media supplemented with 10 mM succinate. Overnight cultures were diluted 1:25 into 5 mL fresh media and grown under the same conditions for 2 hours before splitting. One culture was treated with 2 µg/mL mitomycin C (MMC at a concentration that induces the UmuDAb-DdrR regulon (15)), and both cultures were incubated for 3 hours to induce gene expression consistent with previous studies (11). Total RNA was purified with the Epicentre MasterPure RNA Purification kit. The contaminating DNA was removed with Invitrogen TURBO DNA-*free* and verified by the absence of PCR product using primers specific for *A1S_0636*. Total RNA was measured with the Qubit RNA BR assay (Invitrogen) and samples had values above 6 when measured with the Qubit RNA IQ assay (Invitrogen). 1 µg of total RNA was reverse transcribed with the iScript cDNA Synthesis kit (Bio-Rad) followed by qPCR with iTaq Universal SYBR Green Supermix (Bio-Rad), and RT-qPCR primers were verified for amplification efficiency as previously described (15). Data were analyzed in GraphPad Prism using one-way ANOVA with Dunnett’s multiple comparisons tests as described in the text or each figure legend.

**TABLE 2:** Plasmids and strains used in this study

Plasmid or strain	Description/purpose	Source or reference
Plasmids		
pET-15b	Expression vector; Crb^R^	Novagen
pET15bUDAb	Expression of UmuDAb; Crb^R^	This study
pET15bUDAb-HTH1	Expression of UmuDAb E24K; Crb^R^	This study
pET15bUDAb-HTH2	Expression of UmuDAb K40P R41P; Crb^R^	This study
pET15bUDAb-N	Expression of UmuDAb N100D; Crb^R^	This study
pET15bUDAb-G	Expression of UmuDAb G124D; Crb^R^	This study
pET15bUDAb-W	Expression of UmuDAb W192X; Crb^R^	This study
pET15bUDAb-R	Expression of UmuDAb R201X; Crb^R^	This study
pET15DR	Expression of DdrR; Crb^R^	This study
pEX18Gm	Suicide vector; Gm^R^	(18)
pEX17M	pEX18Gm containing 4 kb genomic fragment of 17,978; Gm^R^	(8) (8)
pEX17M-G124D	pET17M bearing G124D mutation	This study
pEX17M-W192X	pET17M bearing mutations of W192 and G193 codons to stop codons	This study
Strains		
17978 JH	*A. baumannii* ATCC 17978 UN-like; AbaAL44-positive	ATCC
JH1600	*A. baumannii* ATCC 17978 JH Δ*umuDAb*::*kanR*; Km^R^	(15)
JH1700	*A. baumannii* ATCC 17978 JH *ddrR*::TnLK; Km^R^	(2)
DC12	*A. baumannii* ATCC 17978 JH *umuDAb W192X*; produces UmuDAb W192X (12 amino acid C-terminal truncation)	This study
DC13	*A. baumannii* ATCC 17978 JH *umuDAb G124D*; produces UmuDAb G124D	This study

### Mutant strain construction

The Agilent QuikChange II XL mutagenesis kit was used to make unmarked, site-directed mutations in *umuDAb* (*G124D* or *W192X*) contained in plasmid pEX17M, which is a pEX18GM suicide vector containing ~4 kb of 17,978 genomic DNA. Mutagenic primers were those used previously (10). The primers to construct pET17M-W192X introduced two nucleotide changes that modified the codons for W192 and G193 to stop codons. For simplicity, this mutant will be subsequently called UmuDAb W192X. Construction of mutant strains from pET17M-G124D and pET17M-W192X was conducted as in previous work (2) and confirmed with DNA sequencing of PCR products from the putative mutant strains.

## RESULTS

### UmuDAb binds tightly and specifically to its operator in *ddrR-umuAb* promoters

To determine which domains of the atypical UmuDAb repressor were crucial for binding DNA, we first established the binding affinity of native UmuDAb for its wild-type operator. We conducted a series of EMSAs using a 5′-labeled fluorescent probe as a target for recombinant, purified UmuDAb binding (Fig. S1 shows purified proteins). The probe contained the consensus observed for UmuDAb binding previously (5) as well as two eight-bp inverted repeats overlapping the known −35 promoter consensus elements for *ddrR* and *umuDAb* (2). Similarly placed inverted repeats were observed in this region in *Acinetobacter baylyi* sp. ADP1 and predicted to contain a UmuDAb binding site (6). This predicted UmuDAb binding site, defined by the border of these inverted repeats, was flanked by five bp on either end of our probe (Fig. 1).

**Fig 2:**
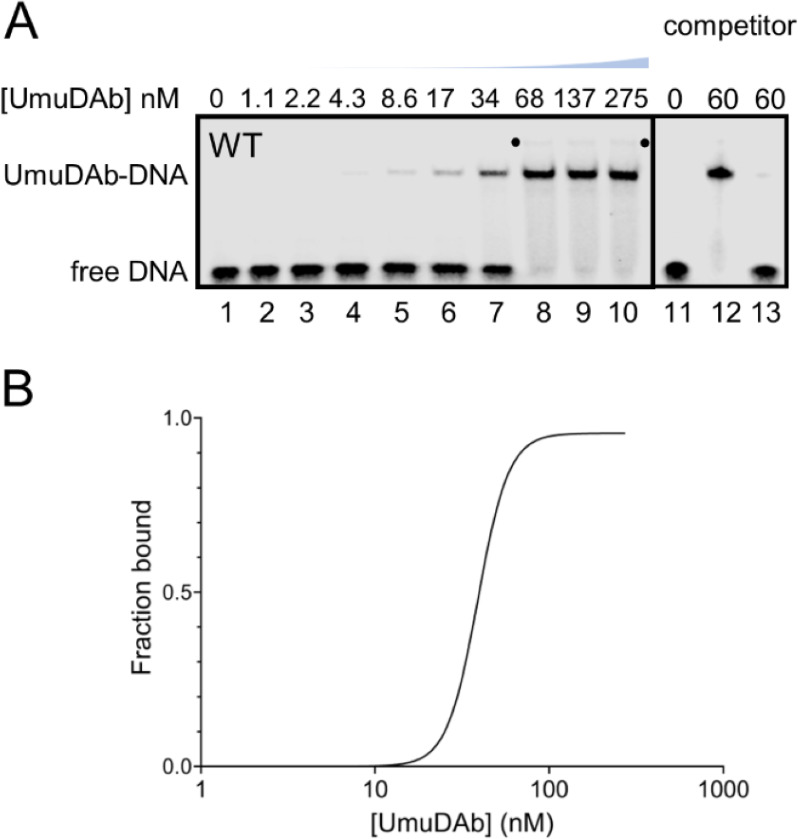
UmuDAb binds to a 37 bp probe sequence in the *umuDAb-ddrR* promoter region. (**A**) UmuDAb bound tightly to DNA in an EMSA. The EMSA was performed with a 5′ IRDye700-labeled probe located 37–73 bps upstream of *umuDAb*. Purified UmuDAb was used in the nM range of concentrations indicated above image in lanes 1–10. The dots highlight a super-shifted band present in lanes 8–10. The small blue graph above the image quantitatively shows the increase in UmuDAb concentration. Lanes 11–13 show that UmuDAb was competed away from the probe. The EMSA was conducted in the absence (lane 11) or presence of 60 nM UmuDAb (lanes 12–13) and 100× of an unlabeled WT competitor sequence (lane 13). **(B**) The best-fit curve of specific binding with Hill slope is shown, based on data averaged from three independent experiments.

These EMSA experiments used a range of UmuDAb concentrations from 1.1 to 275 nM (Fig. 2A, lanes 1–10). These showed one predominant shifted band and one faint higher-molecular weight (MW) shifted band (see a dot in Fig. 1A). We quantitated the fraction of the labeled DNA probe bound by UmuDAb, which increased in a concentration-dependent manner. Fitting the data with non-linear regression with specific binding using the Hill slope yielded an apparent dissociation constant (K_D_) for UmuDAb binding of 38.9 nM (with a 95% confidence interval from 36.5 to 42.1 nM) and a Hill constant of 4.9 (Fig. 2B), which suggested cooperative binding. Ninety-seven percent of this binding was competed away by a 100-fold excess of unlabeled probes (Fig. 2A, lanes 11–13).

### Helix-turn-helix motif mutations slightly reduce UmuDAb binding to its operator

UmuDAb contains an N-terminal putative helix-turn-helix that is not predicted by BLAST or Pfam domain searches, yet is needed for repression of UmuDAb-DdrR repressed genes (9). No causal relationship between DNA binding and the HTH motif has been demonstrated, leading us to directly test whether UmuDAb needed the HTH motif to bind its operator.

**Fig 3:**
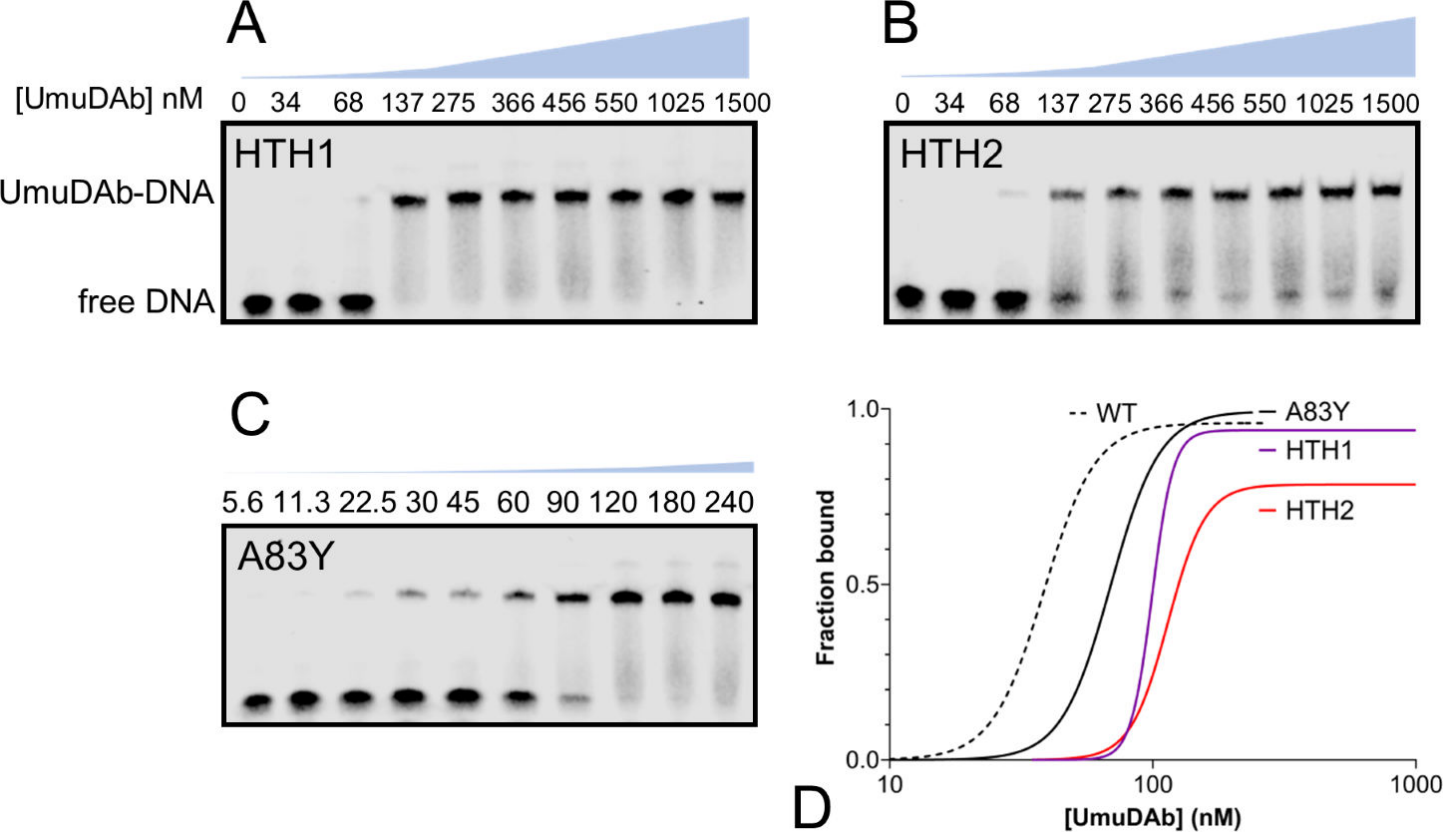
Mutations in UmuDAb HTH moderately affect binding to UmuDAb binding site An EMSA was performed with the wild-type 5′ IRDye700-labeled probe as in Fig. 2. Purified UmuDAb mutants (**A**) HTH1, (**B**) HTH2, or (**C**) self-cleavage mutant A83Y was used in nM range of concentrations indicated above each image. HTH1 and HTH2 mutants displayed similar apparent dissociation constants of K_D_ 99 or 114 nM, respectively, while the self-cleavage mutant bound with a K_D_ of 69 nM, less than twofold increased from wild-type UmuDAb. The blue graph above each image quantitatively represents the increase in UmuDAb concentration at the same vertical scale shown in Fig. 2—note very different scales for panels A and B compared to C. Gels are representative of at least three independent experiments. (**D**) Comparison of the best-fit curves of specific binding with Hill slope, based on data averaged from three independent experiments.

**Fig 4:**
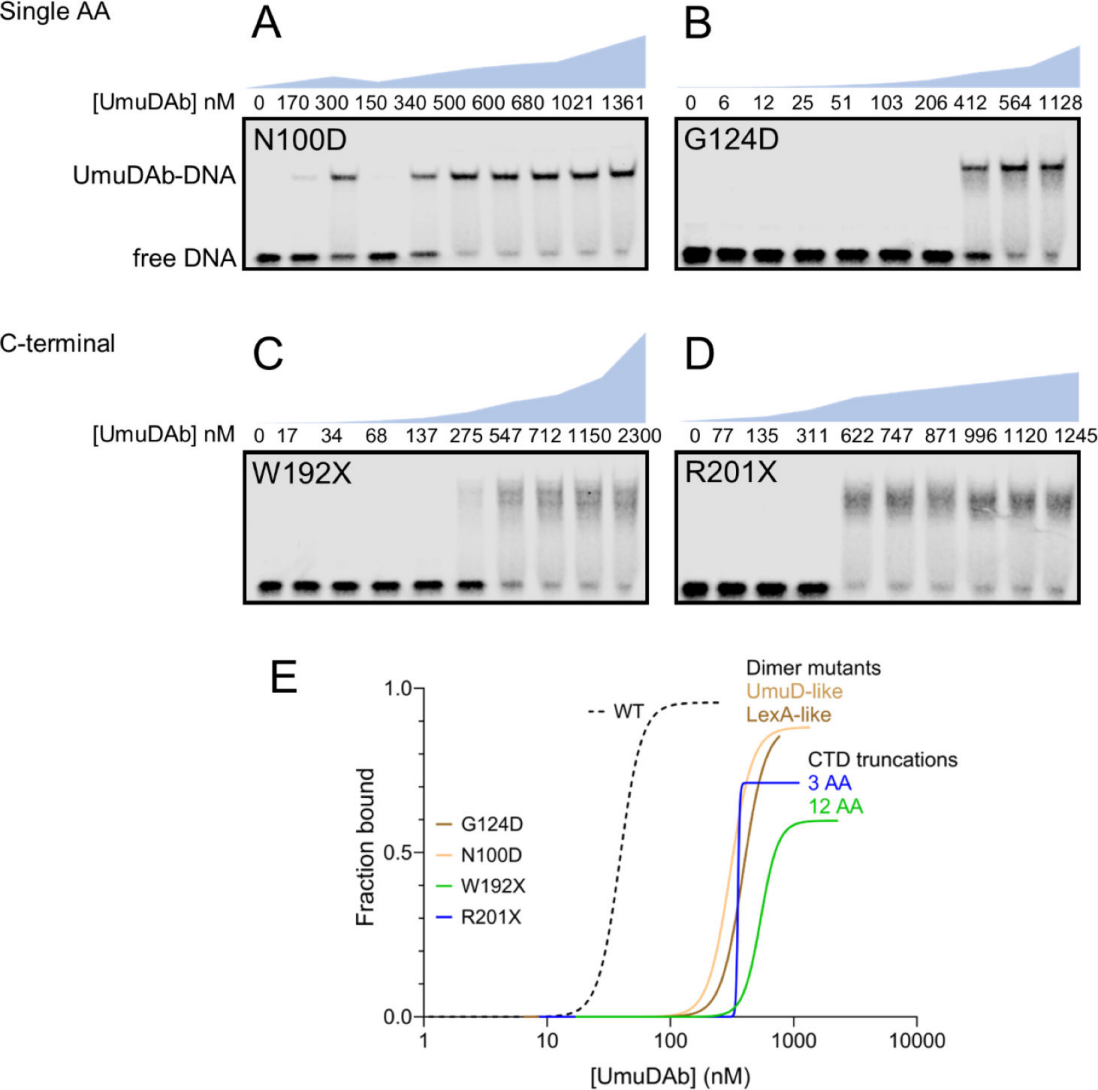
Mutations in UmuDAb residues required for dimerization greatly affect binding to the UmuDAb binding site. An EMSA was performed with the wild-type 5′ IRDye700-labeled probe as in Fig. 2. Purified UmuDAb (**A**) N100D, (**B**) G124D, (**C**) W192X, and (**D**) R201X were used in nM range of concentrations indicated above each image. N100D, G124D, and R201X mutants displayed similar apparent dissociation constants of K_D_ 306, 390, and 353 nM, respectively, while the W192X mutant was more compromised for binding, with a K_D_ of 540 nM. The blue graph above each image quantitatively represents the increase in UmuDAb concentration at the same vertical scale shown in Fig. 2—note very different scales for panel C. Gels are representative of at least three independent experiments. (**E**) Comparison of the best-fit curves of specific binding with Hill slope, based on data averaged from three independent experiments.

We purified UmuDAb proteins bearing HTH mutations in either the first (*HTH1*, conferring an E24K mutation) or second (*HTH2*, substituting helix-disrupting prolines for K40 and N41) helix of the HTH motif. These mutations are known to prevent UmuDAb repression of gene transcription (9), and we used them in EMSAs. Both HTH mutant UmuDAb proteins were impaired for operator binding relative to native UmuDAb (Fig. 3A and B). UmuDAb dilutions used in these representative EMSA images were chosen to collect DNA binding data above and below the apparent K_D_ observed in pilot experiments. A graphic of the UmuDAb concentration is shown above each EMSA image for all WT and mutant proteins in Fig. 2 to 4. Mutations in the second helix (*HTH2*) conferred slightly more impairment for DNA binding, with a K_D_ of 114 nM, than the *HTH1* mutation, which produced a K_D_ of 99 nM. Furthermore, the HTH2 mutant also achieved only 78% binding of the probe at micromolar concentrations (Fig. 3B), while the HTH1 protein bound 94% of the probe (Table S1). Notably, these mutants’ DNA binding cooperativity (as reflected in the Hill constant) increased either slightly (to 6.1 in the HTH1 mutant, from UmuDAb’s 4.9) or more than doubled to 10.8 in the HTH2 mutant (Table S1).

As a control, we also constructed a non-cleavable UmuDAb mutant where the alanine of the AG cleavage site was mutated to tyrosine (11) and assessed its ability to bind DNA. We hypothesized that because LexA cleavage ability is not required for DNA binding, the A83Y protein would resemble native UmuDAb protein binding. The K_D_ of this mutant increased less than twofold over UmuDAb, to 70 nM (Fig. 3C). This was lower than even the least-impaired mutant (HTH1; Fig. 3D). Its Hill constant of 4.65 was unaffected relative to the native protein, indicating no change in the cooperative nature of its binding to DNA.

### Monomeric UmuDAb is very impaired for DNA binding

Because repressors in the S24 serine protease family to which UmuDAb belongs, such as LexA and CI-like prophage repressors, typically form dimers before binding their operators (19, 20), we investigated whether UmuDAb dimer formation was necessary for DNA binding. Previous two-hybrid analyses showed that UmuDAb C-terminal truncations of either 3 or 12 amino acids could not dimerize (10). Dimerization was also significantly (G124D) or mildly (N100D) impaired when a single residue was mutated (10). We, therefore, conducted EMSAs with purified proteins bearing each of these four mutations affecting UmuDAb dimerization (Fig. 4E; Fig. S1).

The recombinant G124D and N100D proteins increased the K_D_ of UmuDAb ~8-fold to 10-fold over native UmuDAb (Fig. 4A and B). The more significant impairment of G124D than N100 in DNA binding (K_D_ of 390 nM vs 306 nM, respectively) mirrored that seen in two-hybrid experiments (10). In addition, neither of these mutants achieved even 90% binding (a B_max_ of 0.90, Table S1) of the probe at micromolar concentrations, unlike the HTH1 or self-cleavage mutants (Fig. 3) or native UmuDAb (Fig. 2B). However, these mutants’ Hill constants did not change (Table S1).

The binding of C-terminal UmuDAb truncation mutants W192X and R201X was unstable, achieving only 60%–70% probe binding at concentrations over 1 µM. They also showed multiple band shifts, especially for W192X. W192X had a 14-fold increase in K_D_ over native UmuDAb (to 540 nM; Fig. 4C), making it more impaired for DNA binding than the single residue mutants G124D or N100D or the smaller, three amino acid truncation R201X, whose K_D_ increased ninefold (to 353 nM; Fig. 4D). The W192X mutant’s Hill constant rose slightly, to 6.1, equaling that of the HTH2 mutant.

### UmuDAb monomers cannot repress the UmuDAb-DdrR regulon

We hypothesized that UmuDAb dimers were required to repress the UmuDAb-DdrR regulon for two reasons. First, UmuDAb dimerization was crucial for DNA binding (Fig. 4). Second, by comparison, HTH UmuDAb mutants cannot repress the UmuDAb-DdrR regulon (8), even though they bind to the operator with a higher apparent K_D_ than monomer mutants G124D and W192X (Fig. 4).

**Fig 5:**
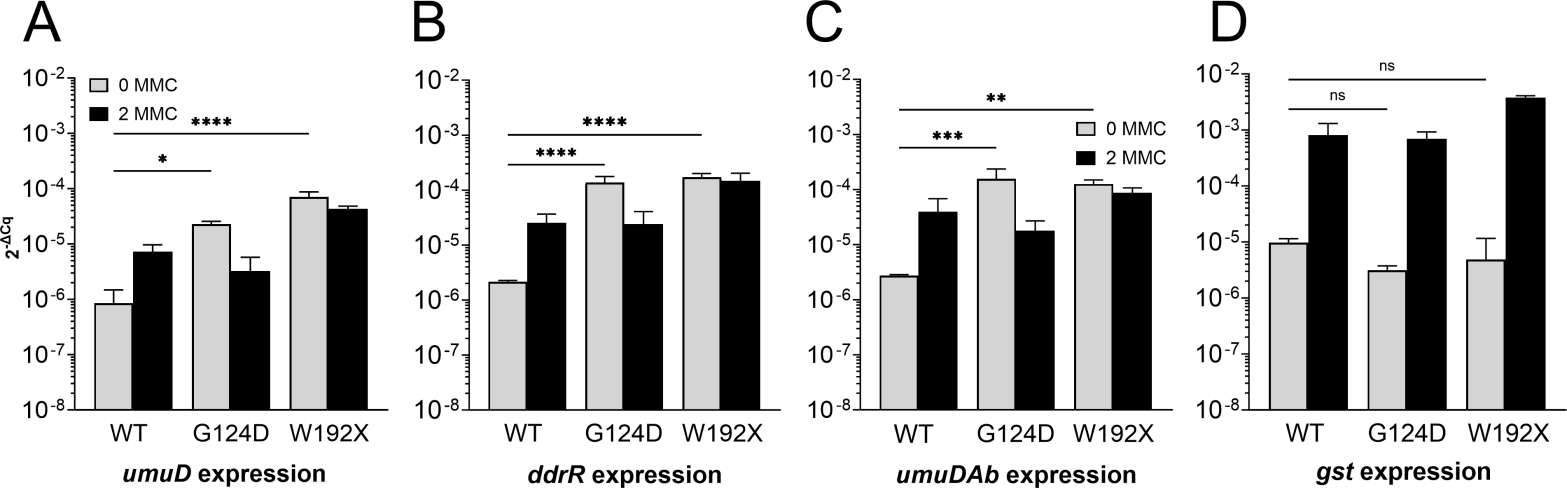
Monomer mutants UmuDAb G124D and W192X cannot repress genes in the UmuDAb-DdrR regulon. Expression of UmuDAb-regulon genes in WT, UmuDAb monomer mutant G124D and C-terminal truncated, monomer mutant UmuDAb W192X strains of *A. baumannii* 17978 JH. RT-qPCR experiments measured gene expression (2^-ΔCq^) in the absence or presence of DNA damage (0 or 2 µg/mL MMC). The expression of representative UmuDAb-repressed genes (**A**) *umuD (A1S_0636),* (**B**) *ddrR (A1S_1388*), (**C**) *umuDAb (A1S_1389*), and (**D**) *gst* (*A1S_0408*) are shown. The UmuDAb monomer mutants produced higher expression than WT cells in the absence of DNA damage (one-way ANOVA test with Dunnett’s multiple-comparison post-testing). However, (**D**) the DNA damage-inducible but non-UmuDAb-regulated *gst* gene (15) was still repressed in the absence of DNA damage in the *G124D* and *W192X* mutant strains. The standard deviation of the means from technical triplicates tof biological triplicates is shown. ****P < 0.0001; ***P < 0.001; **P < 0.01; *P < 0.05. ns = not significant.

This hypothesis was tested *in vivo* using strains DC13 and DC12 that bear markerless *umuDAb G124D* or *umuDAb W192X* mutations, respectively, in the chromosome of 17978 JH. We subjected these strains to MMC DNA damage, which induces the UmuDAb-DdrR regulon (15), and measured the transcription of UmuDAb-repressed genes in the presence and absence of MMC. In the absence of MMC, DC12 and DC13 showed significant derepression of the two chromosomal genes (*ddrR* and *umuDAb*) and one plasmid-based gene (*umuD A1S_0636*) that we tested (Fig. 5), consistent with the hypothesized inability of the G124D and W192X UmuDAb mutant proteins to bind DNA and repress transcription. By contrast, the *gst* (*A1S_0408*) gene, which is induced by DNA damage but not repressed by UmuDAb-DdrR, maintained its repression in DC12 and DC13 cells in the absence of MMC but was (15) induced after MMC treatment (Fig. 5D). This suggests that the deficiency in DNA binding of monomeric UmuDAb forms did not interfere with non-UmuDAb-repressed gene expression.

### DdrR modestly enhances UmuDAb binding

**Fig 6:**
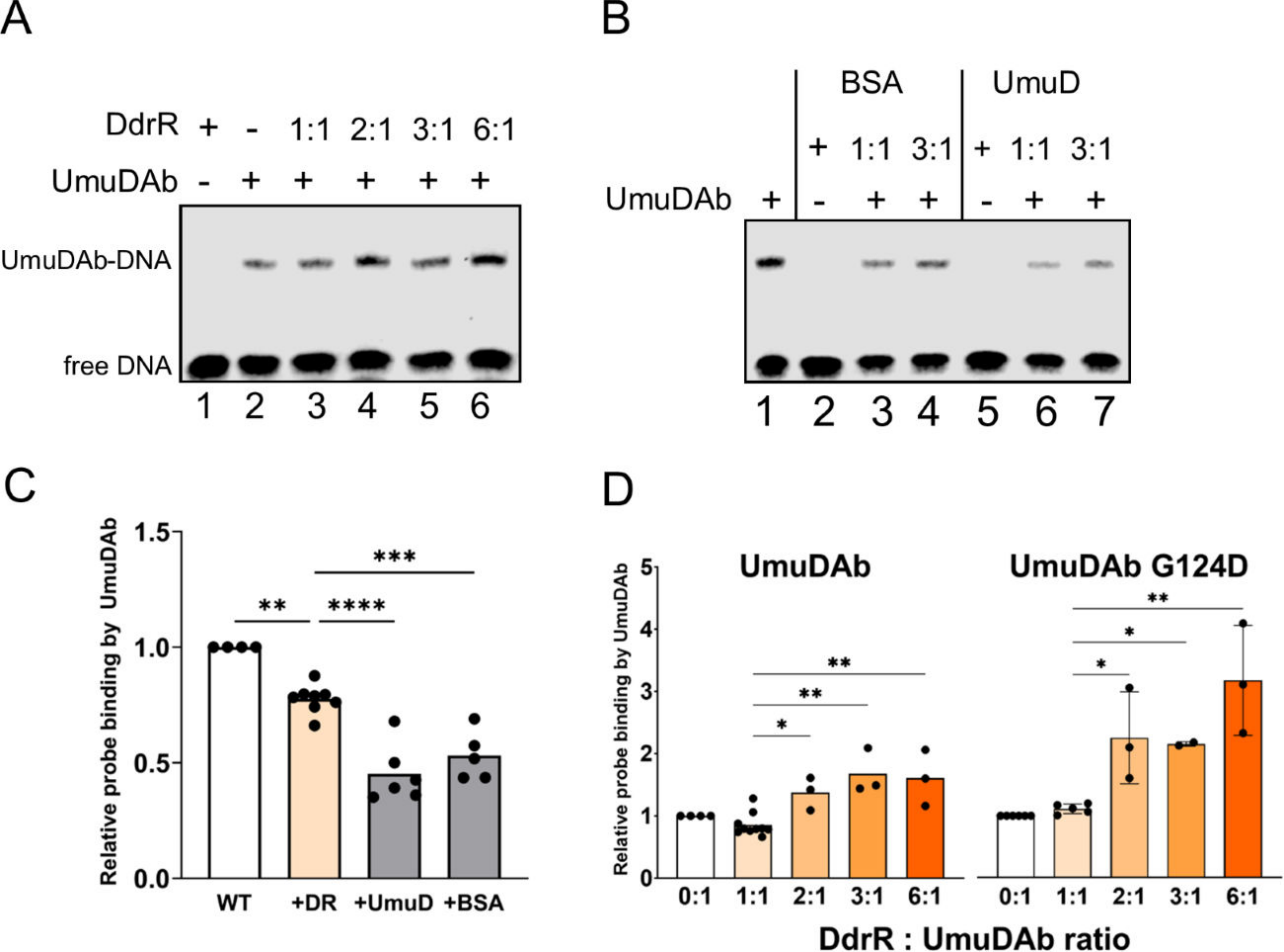
DdrR enhances UmuDAb binding to its operator only when present in greater than equimolar concentrations EMSAs were conducted with wild-type labeled probe and 20 nM UmuDAb, in the absence (−) or presence (+) of equimolar or greater (2:1, 3:1, 6:1) of (**A**) DdrR, (**B**) *E. coli* UmuD, or BSA added to the binding reaction. Negative controls UmuD and BSA (B, lanes 2, 5), like DdrR (A, lane 1), did not bind DNA. (**C, D**) The fraction of labeled probe bound to WT UmuDAb alone or UmuDAb and a second protein (DdrR, UmuD, or BSA) was quantified and analyzed with LI-COR ImageStudio 5.2 software. (**C**) UmuDAb and DdrR (DR), UmuD, or BSA at 1:1 molar ratios show that UmuD and BSA reduce UmuDAb binding relative to DdrR, which had lower binding that UmuDAb in the absence of DdrR. (**D**) The binding of both UmuDAb and monomer mutant UmuDAb G124D increases with greater than equimolar ratios of DdrR to UmuDAb. The fraction of UmuDAb binding in the absence of a second protein (0:1) was normalized to 1.0. For panels C and D, the data shown are averages from 4 to 5 independent samples for all conditions except WT + DR, which was tested in 10 experiments. Significant differences are shown after one-way ANOVA with Dunnett’s multiple comparison post-testing. ****P < 0.0001; ***P < 0.001; **P < 0.01; *P < 0.05.

The corepressor DdrR enhances UmuDAb repression by an unknown mechanism (8). We hypothesized that DdrR helps UmuDAb bind DNA, so to measure the effects of DdrR, we added purified DdrR to a concentration of UmuDAb that yielded modest (10%–15%) binding in an EMSA binding reaction. As previously observed (3), DdrR alone did not bind to the DNA probe (Fig. 6A, lane 1). Surprisingly, adding an equimolar amount of DdrR did not increase UmuDAb binding to its operator, (Fig. 6A, lane 3), but slightly decreased it (Dunnett’s multiple comparisons testing after one-way ANOVA, Fig. 6C). However, evidence suggests that DdrR can form homodimers (10), so we used greater amounts of DdrR in additional EMSAs. When we used 2:1, 3:1, or 6:1 ratios of DdrR to UmuDAb, modest but significant increases (1.6- to 1.9-fold, with one-way ANOVA and Dunnett’s multiple comparisons testing) to UmuDAb binding were observed (Fig. 6D, first five bars). Similarly, increasing ratios of DdrR to the monomer UmuDAb G124D protein yielded even greater increases in the fractions of the probe bound by this UmuDAb mutant (Fig. 6D, last five bars).

As controls, we added bovine serum albumin (BSA) or UmuD proteins in equimolar or greater concentrations to UmuDAb instead of DdrR. They did not bind DNA or increase UmuDAb binding to the probe (Fig. 6B). While the fraction of DNA bound by a 3:1 ratio of DdrR to UmuDAb was twofold greater than a 1:1 ratio, BSA showed only a 1.15-fold greater fraction of binding at 3:1 than 1:1, and UmuD had the same fraction bound at a 3:1 ratio as a 1:1 ratio. Furthermore, even at a 1:1 ratio, DdrR + UmuDAb displayed a higher fraction of probe binding than either BSA + UmuDAb or UmuD + UmuDAb (Dunnett’s multiple comparisons testing after one-way ANOVA) (Fig. 6C).

### The UmuDAb operator’s core is more important than the inverted repeats flanking it

**Fig 7:**
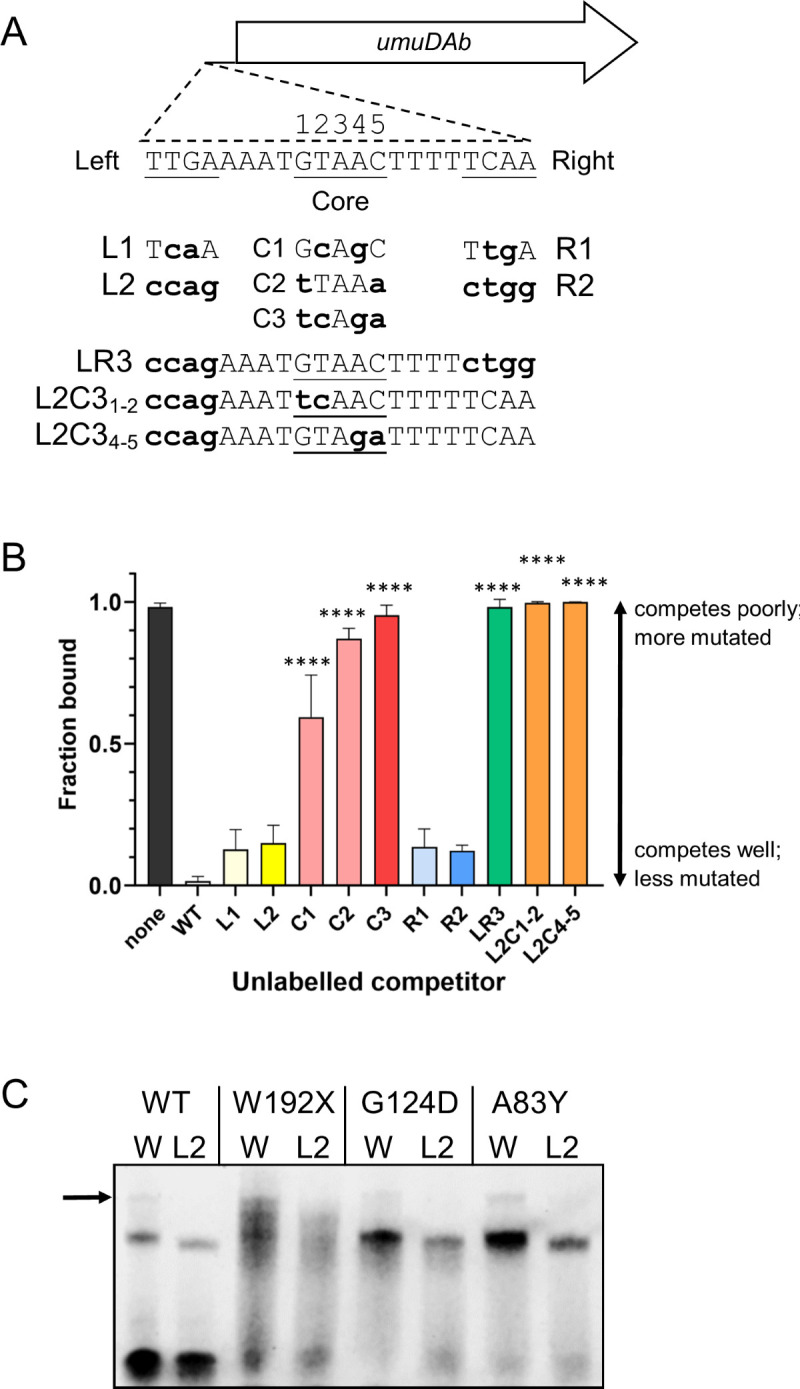
Mutations to the UmuDAb operator affect UmuDAb binding (**A**) Diagram of the 21 base pair UmuDAb operator upstream of *umuDAb*. The UmuDAb binding consensus sequence with the required sequence elements [the four base pair left and right arms (inverted repeats) and the five-bp central core] underlined. Mutations to the 37 bp probe sequence shown in Fig. 1 were made in a set of 10 competitor oligonucleotides that were then used in EMSA experiments. Bold lowercase font denotes mutations to either the left arm (L1, L2), right arm (R1, R2), or core (C1-C3) region (base pairs not shown are present and wild-type sequence). Also shown are competitor oligonucleotides with both arms (LR3) or combinations of mutations to L2 and C3 bases (L2C1-2 and L2C4-5). (**B**) Fraction of labeled probe bound to wild-type UmuDAb in control conditions (“none”) or the presence of 100× of unlabeled WT or mutant competitor oligonucleotides. Competitor oligonucleotides that allowed a large fraction of the labeled probe to bind are poor competitors and consist of more mutated UmuDAb binding sites, and vice versa. Increasing color saturation indicates the relative number of mutations present in the competitor, and the compound bar colors green and orange represent a combination of mutated sequences. Significant differences from the fraction bound in the presence of WT competitor (one-way ANOVA with Dunnett’s multiple comparison post-tests) are shown, with four asterisks designating adjusted *P* < 0.0001. Unlabeled comparisons (L1, L2, R1, and R2) had adjusted *P* > 0.05. (**C**) EMSA comparing binding of UmuDAb WT and W192X, G124D, or control A83Y mutant proteins to labeled wild-type (“W”) or left-arm mutated (“L2”) probes. A weaker, higher molecular weight band (arrow) above the predominant band was observed only with the WT probe, regardless of the WT or mutant UmuDAb form used.

To quantitatively assess and compare the roles of the inverted repeats and the core of the UmuDAb operator, we designed 10 oligonucleotide probes that contained mutations in the left arm, right arm, and/or the core (Table 1). EMSA analyses were performed using a 100-fold excess of these mutant, unlabeled probes (Fig. 7A) as competitors to the labeled wild-type operator. Positive control binding reactions containing no competitor showed 98%–100% of the labeled wild-type probe binding to UmuDAb. By contrast, when a wild-type (WT) operator was used as a competitor, UmuDAb binding to the labeled probe was essentially abolished (<2% bound) (Fig. 7B). We observed an inverse binding relationship where mutated competitors that were less capable of binding UmuDAb allowed more labeled probe to be bound by UmuDAb.

Mutation of either two or all four of the left or right arm base pairs (L1, L2, R1, and R2; Fig. 3A), yielded effective competitors resulting in low amounts (~12%–15%) of labeled wild-type probe binding that were not different than an unlabeled wild-type competitor (Fig. 7B). However, mutating both the left and right arms (competitor LR3) to create a different palindrome (CCAG-N_13_-CTGG) completely prevented LR3 from competing with the labeled wild-type probe (Fig. 7B). This suggested that at least one arm was required for UmuDAb binding, but not both, and that the specific content of the palindrome was crucial for UmuDAb binding. This was consistent with similar experiments conducted with a labeled competitor bearing the L2 mutation, which did not increase the K_D_ relative to the labeled wild-type probe (Fig. S2).

To test the role of the core, we used competitors mutated in the GTAAC sequence to GcAgC (C1), tTAAa (C2), or tcAga (C3) (Fig. 7A; lowercase bases are mutations). These mutations maintained a palindrome to test whether the specific palindrome bases were important or if any palindrome would allow UmuDAb binding. Mutation of any two base pairs significantly decreased UmuDAb binding, and mutation of all four base pairs to form a new palindrome (in C3) abolished DNA binding (Fig. 7B). ANOVA analyses showed that mutating the entire core region in C3 was more detrimental to UmuDAb binding than mutating either the right or left arm (R1, R2, L1, or L2), and was as detrimental as simultaneously mutating both the left and right inverted repeats (in LR3) (adjusted *P* < 0.0001).

We next designed oligonucleotides combining the left arm L2 mutation with either the left-most (bases 1–2) or right-most two (bases 4–5) of the core (Fig. 3A, competitors L2C3_1-2_ and L2C3_4-5_). These oligonucleotides failed to compete with labeled wild-type (Fig. 7B) or L2-mutated (Fig. S3) labeled DNA probes. These results suggested that the mutation of only two core bases was sufficient to prevent competition with the WT-labeled probe. In addition, the greater effect of removing the palindrome in L2C3_1-2_ and L2C3_4-5_ compared to palindromic competitors C1 and C2, which were also mutated in two core bases, indicated the importance of a palindrome to effective DNA binding.

Finally, we tested whether this large, 21-base pair operator was composed of two half-sites [e.g., comprising either the left (or right) arm and the core], or if the entire sequence contained only one binding site. We conducted EMSAs with labeled WT or L2 mutant probes and observed that in addition to the predominant shifted DNA band, a higher MW band shift was present when the WT, but not the left arm mutated probe L2-labeled probe, was used (Fig. 7C; see Fig. 2A). This suggested that two binding sites for UmuDAb were present in the operator. The ability of monomers W192X and G124D to bind to the WT, but not the L2-labeled probe, is consistent with this interpretation.

## DISCUSSION

### Relative importance of UmuDAb protein motifs in binding DNA

We have previously identified UmuDAb motifs required for its dimerization (10) and gene repression actions (2, 9), but no one has tested what is directly required for its DNA binding ability. LexA is known to recognize its operator as a dimer (19) with the second helix of its HTH motif making major groove contacts that are sequence-dependent (7, 21). This work showed that both helices 1 and 2 of the putative HTH motif in UmuDAb affect UmuDAb binding to DNA, with disruption of helix 2 raising the apparent binding coefficient slightly more than disruptions in helix 1, suggesting that HTH2 may be more crucial to DNA binding and repression of transcription than HTH1. These relative contributions to DNA binding match what we saw in these mutants’ ability to repress transcription: mutations to HTH1 slightly impaired repression, but HTH2 mutations nearly completely inhibited repression of transcription (8). As a comparison, the control non-cleavable A83Y mutant UmuDAb protein showed only mild impairment (<2-fold increase in K_D_) of binding to the UmuDAb operator. Although this is a small increase, it could reflect an overall perturbation of the UmuDAb structure due to the large tryptophan substitution at the alanine of the Ala83-Gly84 site.

**Fig 8:**
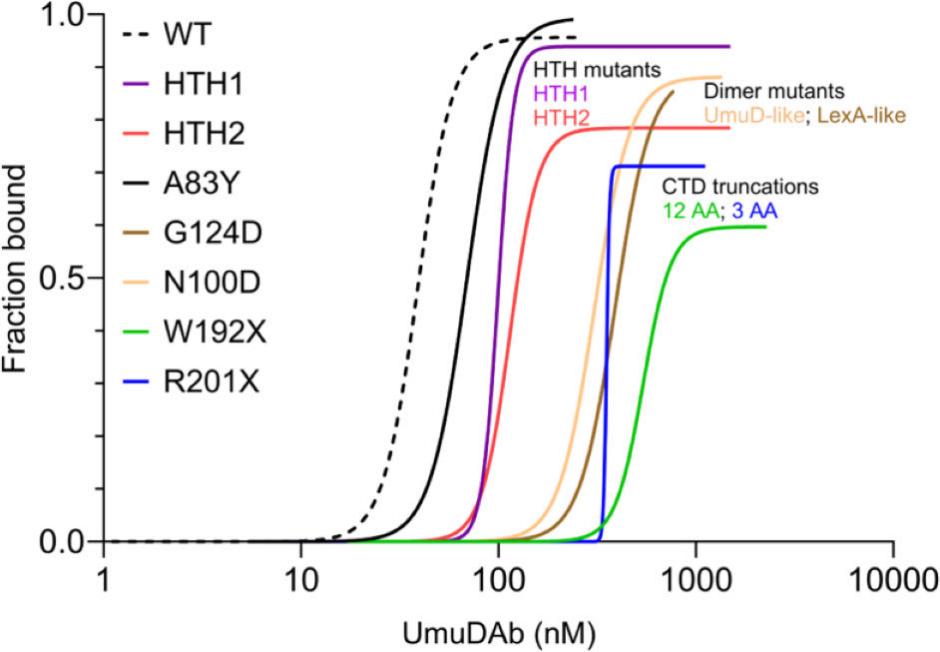
Comparison of WT UmuDAb and all mutant forms’ best-fit curves of specific binding with Hill slope, based on data averaged from three independent experiments.

We also tested the effects of either point or truncation mutations in UmuDAb that decreased dimerization in previous two-hybrid assays (10). In this study, point mutation of the G124 residue that is required for LexA and UmuDAb dimerization nearly abolished DNA binding, while point mutation of the N100 residue, only modestly important for UmuD dimerization, was moderately important for DNA binding (Fig. 4A and B). By contrast, C-terminal truncations increased the apparent K_D_ of UmuDAb for its operator more than the G124D and N100D point mutations, and the larger (12 AA) truncation W192X destabilized and affected DNA binding more strongly than the three AA R201X truncation. Thus, the order of importance of the UmuDAb regions for dimerization in two-hybrid assays is consistent with what we observed for DNA binding: the CTD, then G124, then N100. These results imply a model of UmuDAb action like LexA and phage repressors, with dimerization being a major factor in binding DNA (22, 23). Figure 8 shows the impact of each mutation on DNA binding, relative to WT UmuDAb.

Because UmuDAb G124D and W192X dimerization mutant proteins bound the operator more poorly than the HTH1 and HTH2 mutants, we hypothesized that G124D and W192X mutant strains would show greater derepression of gene expression than in the HTH1 and HTH2 mutant strains. However, although UmuDAb G124D and W192X monomers did not repress expression of either chromosomal or plasmid-based members of the UmuDAb-DdrR regulon (Fig. 5), the regulon was repressed less than the HTH2 mutant, and only more derepressed than the HTH1 mutant. Hence, it seems that apparent binding affinities observed in EMSAs with such poor affinity as G124D, and even more so, the C-terminal truncation mutants W192X and R201X, must be carefully interpreted for *in vivo* implications, as RT-qPCR results for the G124D mutant indicated completely ineffective gene repression. Our data indicate a complex relationship between *in vitro* DNA binding and *in vivo* gene repression where a threshold of DNA binding ability (i.e., K_D_) may exist for UmuDAb to maintain repression.

One way to interpret the biological significance of these deficiencies in DNA binding is to compare the K_D_ increases of the UmuDAb mutants to the levels of UmuDAb in cells. While the actual concentration of UmuDAb is unknown, we and others have observed that *umuDAb* expression is moderately induced by DNA damage ~3.5- to 4-fold (5, 15). By comparison to LexA studies, which show 600–800 molecules per cell in the uninduced state (24), it is possible that a similar number of UmuDAb repressor molecules exist. However, the UmuDAb-repressed regulon is smaller than LexA (5, 15), perhaps necessitating fewer UmuDAb molecules per cell. We found that *HTH1* and *HTH2* mutations significantly disrupted UmuDAb-mediated gene repression *in vivo* (8) even though their K_D_ only increased 2.5- to 3-fold in this study, again suggesting that slight differences in UmuDAb concentrations may affect repression. By contrast, the much larger increases in K_D_ (7.9- to 13.9-fold) observed for the four monomer mutants (Table S1) suggest that even the 3.5- to 4-fold increased UmuDAb concentration after DNA damage would not be able to compensate for the loss of DNA binding.

### DdrR enhancement of UmuDAb binding

We previously established that DdrR enhances the repression actions of UmuDAb, not its cleavage (8). An outstanding question, therefore, is as follows: how does DdrR enhance UmuDAb repression? Given that operator binding is required for repressors to inhibit transcription, we hypothesized that adding DdrR to a UmuDAb-DNA binding reaction would enhance UmuDAb binding. We saw that equimolar ratios of DdrR did not enhance UmuDAb binding to the probe, but DdrR at 2:1, 3:1, or 6:1 molar ratios increased UmuDAb binding almost twofold (Fig. 6D), supporting our hypothesis. These results are consistent with the actions of gp7, another small corepressor protein that binds as a dimer of dimers to LexA in *B. thuringiensis* and increases LexA DNA binding by approximately twofold (4). However, whereas gp7 also prevents auto-cleavage of LexA, DdrR was found not to prevent activated-RecA mediated UmuDAb auto-cleavage (3). In addition, the differential expression of *ddrR* and *umuDAb* relative to each other supports a biological role for higher levels of DdrR than UmuDAb: *A. baumannii* cells have evolved to express *ddrR* at higher levels after DNA damage (13.7-fold induction, the second most highly induced gene regulated by UmuDAb) than *umuDAb* (3.8-fold induction) (15). Furthermore, our observations that slight increases in K_D_ (in the HTH1 and HTH2 mutants, Fig. 3) were sufficient to relieve repression *in vivo* (8) suggest that a ~2-fold increase in binding could be biologically relevant.

Finally, because dimerization is important for repressor binding to DNA, we speculate that DdrR’s enhancement of UmuDAb binding activity may be through stabilizing or encouraging UmuDAb dimerization. This is supported by seeing a greater effect from adding DdrR to the dimerization-deficient UmuDAb G124D mutant than to WT UmuDAb (Fig. 6D). Although UmuDAb and DdrR bind to each other in SPR experiments (3), and gp7 causes a further supershift in the LexA-DNA probe binding pattern (25), our EMSAs did not show a supershifted probe-UmuDAb complex in the presence of DdrR. However, our DdrR-UmuDAb EMSAs were designed to occur at minimal UmuDAb binding levels to better show enhancement of this activity, whereas we only observed super-shifting of UmuDAb to the probe when the majority of the probe was bound (Fig. 2A). Future experiments using different levels of DdrR expression could test whether increases in DdrR ratios to UmuDAb result in altered repression *in vivo*.

### UmuDAb operator features unique to *Acinetobacter*

Typically, SOS boxes of enteric bacteria contain two short palindromes separated by ~8–10 A-T-rich base pairs, for example, CTGT-N_8_-CAGT box (26, 27) in *E. coli*. Although significant variation exists between LexA/DinR box consensus sequences within Gram-positive and Gram-negative bacteria, the sequences often exhibit similarity between related bacterial species (28, 29). As the Gram-negative *Acinetobacter* genus is not an enteric bacterium like *E. coli*, its different SOS box sequence is to be expected. However, earlier studies indicated not just different nucleotides in a palindrome, but also an unusual structure to the *A. baumannii* UmuDAb binding site (6). As defined by transcriptomic studies (5, 30), it contains a five-base pair core element in the middle of an eight base pair A-T-rich region that separates two flanking TTGA/TCAA sequences that form a palindrome (“arms”; Fig. 1) (5). None of the currently studied LexA boxes contain the unique core structure seen in the UmuDAb operator. Even the non-canonical SOS recognition sites identified in Bacteroidetes more closely match the LexA motif of palindromes flanking a central spacer region, as mutations in the palindromes eliminated binding ability but mutations in the small four-base pair spacer region had no effect (31). When all bases of either arm or core were mutated to eliminate the arms’ palindromic nature, one study saw no UmuDAb binding at 50 nM, but did not determine whether the contributions of the core or the arms were more important to UmuDAb binding, or whether the specific content of the palindromes was required (5). Because the UmuDAb binding sites in the other members of the UmuDAb-DdrR regulon are identical (to Fig. 1) in the left and right arms and the core (GTnAC) (5), we did not repeat these studies with labeled probes to those genes’ consensus sites.

In EMSAs using 10 unlabeled competitor oligonucleotides mutated in the arms or core, we saw that the core was more important for binding than the left or right arms, as the mutated core probes could not compete with the WT probe, but the mutated arm probes could compete. In addition, while both arms were not required (Fig. S1), at least one intact arm was needed to bind DNA, as the LR3 competitor, mutated in both the left and right arms, could not compete with the labeled WT probe for binding. These results are consistent with a surface plasmon resonance study, which qualitatively showed that mutation of either arm modestly affected UmuDAb binding (3). However, another study’s EMSA found that mutation of either arm resulted in total loss of UmuDAb binding (5), which is in contrast to our work, although the conditions of these experiments were quite different in labeling method, probe size (100 bp vs 37 bp) and crucially, amount of UmuDAb used.

Testing for the requirement of a specific operator sequence vs any general palindrome, we found that the specific nucleotides in the UmuDAb operator’s GTnAC core were crucial, unlike the palindrome, either arm of which could have its palindromic nature destroyed with little loss of binding (in competitors L2 and R2). This is consistent with a qualitative study that mutated all core bases while maintaining a palindromic symmetry, like competitor C3, but changed each base from purine to pyrimidine (or vice versa) and saw no DNA binding (5). Our study showed that a specific palindrome was also required in the arms, as a different palindrome (in competitor LR3) was ineffective at competing with the wild-type operator. This requirement for a palindrome is similar to the LexA operators found in *E. coli* (7) and *B. subtilis* (32, 33), although in the LexA SOS boxes, the palindrome is in the arms, because no palindromic core exists.

While the lack of overall palindromic nature to the left arm and core (TGAA(N_4_)GTAAC) or core-right arm (GTAAC(N_4_)TCAA) of UmuDAb operators does not obviously suggest the notion of two adjacent or overlapping operator sites, a similar non-palindromic arrangement has been observed in *B. thuringiensis*, which uses phage protein gp7 to help bind to one of the two adjacent LexA binding sites: palindromic site, *dinBox1,* and an adjacent non-canonical half-site, *dinBox1b* (4). Unlike UmuDAb, LexA binds independently and noncooperatively to both dinBox1 sites (4). Not all SOS boxes are perfectly palindromic (34) and not all differences directly relate to gene regulation (26).

Our results of UmuDAb DNA binding (Fig. 7) are more consistent with a model of two adjacent operator sites in the *umuDAb-ddrR* promoter region (composed of the core and one arm) than one large binding site. For example, either arm was dispensable for wild-type operator-like binding levels, but at least one arm, along with the core, was required. Further support for this model is our observation of two shifted probe-protein bands in the EMSA assays (Fig. 7C). The lower MW band was dominant, and, consistent with other repressors’ behaviors, likely represents one UmuDAb protein bound to the probe, with the higher MW, the fainter band likely composed of two UmuDAb proteins bound to the probe (4, 35). The monomeric mutants UmuDAb G124D and W192X also produced this pattern of two bands (Fig. 7C), suggesting a monomer bound to each operator site. We do not think that the low MW band is composed of two monomeric UmuDAb proteins binding at the two sites because binding to the mutated labeled L2 probe (containing only one site) also produced a gel-shifted band at this position (Fig. S2). Multiple, higher MW (than the dominant) bands were produced by the W192X monomer mutant, further indicating that multiple UmuDAb monomers can bind, albeit with much lower affinity (Fig. 4).

There are precedents for multiple operator sites for DNA damage response genes. SOS genes typically contain a single binding site, but ~75% of sequenced colicin genes (36) and a few *E. coli* DNA damage-response genes (26) contain more than one. Somewhat similar to the UmuDAb binding site, a consensus enteric colicin operator contains a complex LexA binding site, with two overlapping SOS boxes structured as: left arm_5_(N_8_)core(N_8_)right arm_4_ (36). The distal (left) site is more conserved with typical SOS boxes while the proximal binding site differs more, but LexA still cooperatively binds to both boxes at the same concentrations (36, 37). That this consensus is drawn from colicin (toxin) bacterial operators echoes the conclusion that needing to control mutagenic polymerase expression tightly helps explain the collaboration of co-repressors DdrR and UmuDAb (38), as well as other colicin systems employing dual repressors (39). In *E. coli*, only four of the 31 SOS promoters contain multiple non-overlapping binding sites: *recN* (three sites)*, umuDC* (two sites)*, lexA/dinF* (three sites)*,* and *yjdM* (two sites) (26). It is also common for repressors binding multiple sites to do so cooperatively. For example, the dimeric CI phage repressor of phage TP901-1 exhibits cooperative binding, by binding its operators as dimers and higher multimers (22). The ϕKO2 CB repressor has three operator sites, O_R_1, O_R_2, and O_R_3, and can bind all three sites, with the strongest binding affinity to site O_R_1 (23).

This investigation into the atypical UmuDAb repressor and its non-canonical, overlapping binding site was not exhaustive. Additional experiments will be required to elucidate the mechanisms and evolutionary history of this complex regulatory site. Nevertheless, the results from several prior studies, combined with this work, suggest that the UmuDAb binding site functions as an operator for the repression of transcription of the UmuDAb-DdrR regulon. First, in *A. baylyi* strain ADP1, mutation of base 5 of the core and base 1 of the right arm (in strain JH104), the entire right arm (strain JH101), or bases 2 and 4 of the core that maintain the same palindrome as mutant competitor C1 (strain JH103), abolished repression for all members of this regulon (9). Second, mutation of either HTH1 or HTH2 to form mutant UmuDAb proteins, which were compromised for binding (Fig. 3), abolished repression in *A. baumannii* strains MF1 and MF2 (8). Lastly, the monomer UmuDAb G124D, which was very deficient for binding (Fig. 4B), also could not repress transcription.

In this study, we have built upon previous studies that defined the consensus UmuDAb binding site (5, 30) and the required protein motifs for UmuDAb actions (8) in *Acinetobacter baumannii*, using both dimer-competent and monomer UmuDAb proteins as well as mutated operator probes to investigate the requirements for UmuDAb binding to its operator. We have shown the importance of the unique binding site core that is not a true half-site palindrome, and that it is more important for binding than either palindromic arm. We revealed that UmuDAb dimerization is more important than the N-terminal helices for binding to its operator. Lastly, we showed that adding DdrR increased operator binding for UmuDAb native and monomer mutants. The combination of UmuDAb possessing an unconventional HTH-like structure and working with a corepressor protein to bind to a large operator presents a significant deviation from the LexA regulatory mechanism. This information could help investigate DNA damage-induced regulatory networks in other non-canonical damage response pathways.

## SUPPLEMENTAL MATERIAL

Supplemental material jb.00432-23-s0001.pdf Figures S1 to S3 and Table S1.

ASM does not own the copyrights to Supplemental Material that may be linked to, or accessed through, an article. The authors have granted ASM a non-exclusive, world-wide license to publish the Supplemental Material files. Please contact the corresponding author directly for reuse.
