# Supplementary material for: Repression of Acinetobacter baumannii DNA damage response requires DdrR-assisted binding of UmuDAb dimers to atypical SOS box
Source: J Bacteriol. 2024 May 10;206(6):e00432-23. doi: 10.1128/jb.00432-23 (PMC11332147; doi:10.1128/jb.00432-23)
Supplement: Supplemental material — Figures S1 to S3 and Table S1. [file jb.00432-23-s0001.pdf]

Supplemental Figures S1-3 and Table S1

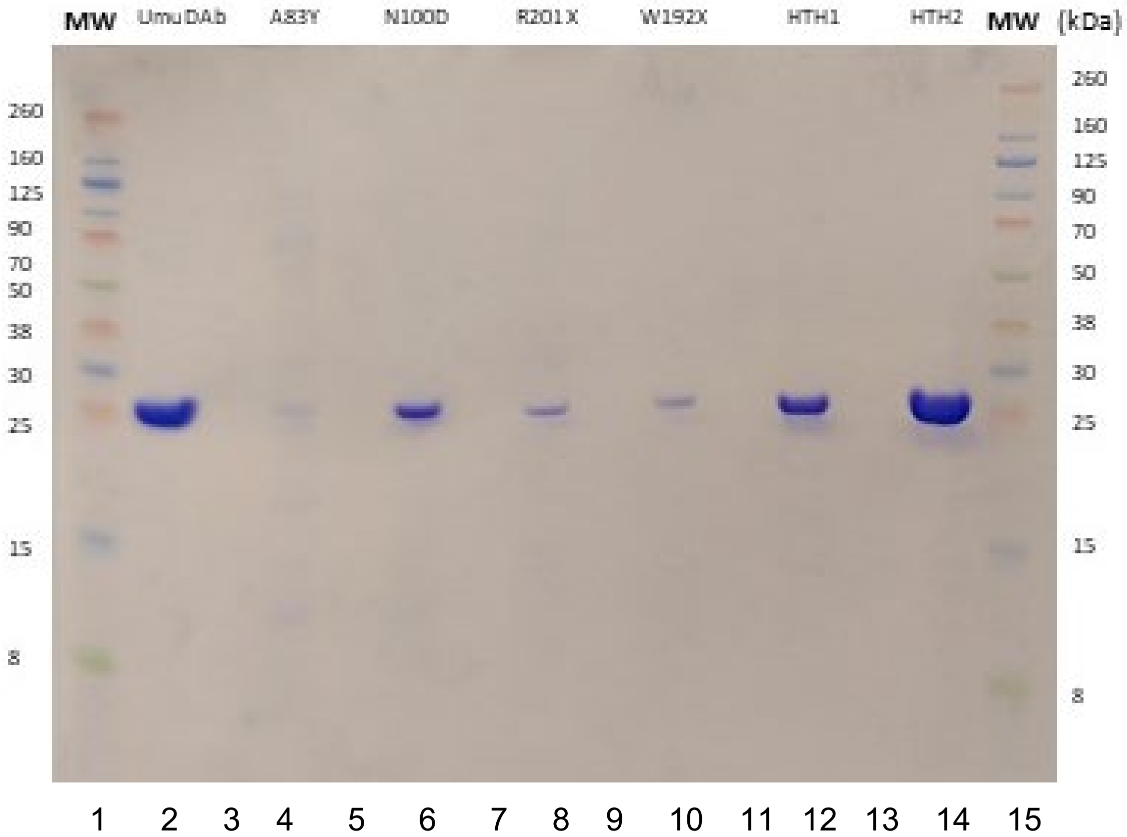

Supplemental Fig 1. Coomassie stain of purified 17978 UmuDAb and mutant UmuDAb proteins. Lanes 1 and 15 contain Chameleon Duo molecular weight markers. Lanes 2 through 14 contain proteins in even-numbered lanes, with odd lanes remaining unloaded (blank). The wildtype protein is labeled UmuDAb. Mutants are labeled by their altered designation. Protein lanes were each loaded with 2 micrograms of purified protein as measured by Qubit.

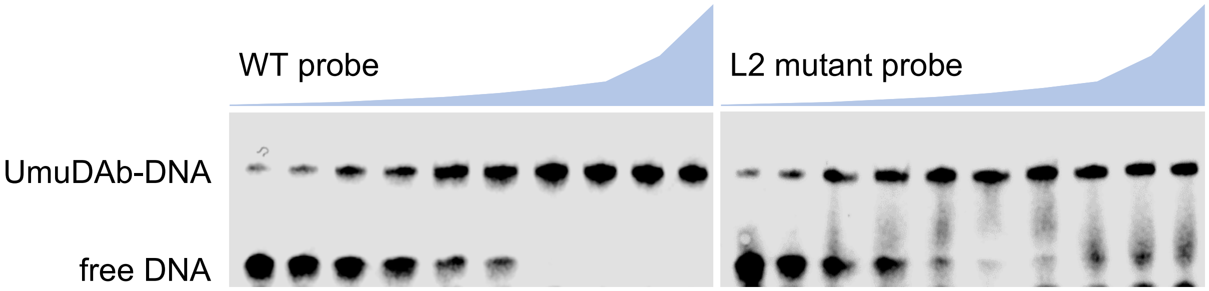

Supplemental Fig 2. EMSAs using WT UmuDAb and either WT labeled DNA or left arm L2 mutation labeled DNA, run on the same day in the same electrophoresis run. UmuDAb dilution preparations and concentrations were the same for both gels; starting at left: 4.3, 8.6, 12.5, 17.1, 25, 34.25, 50, 58.5, 137, 275 nM. The apparent K_D calculated for the L2 mutant probe in this experiment was ~75% of that calculated for the WT probe.

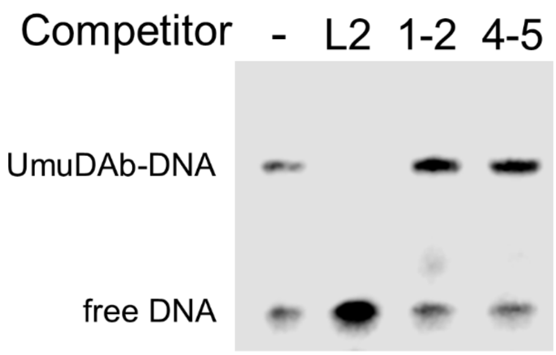

Supplemental Fig 3. EMSA using WT UmuDAb at 60 nM and WT labeled DNA. Unlabeled competitors L2, $L2C3_{1-2}$ ("1-2"), or $L2C3_{4-5}$ ("4-5") were used, showing that mutation of either bases 1-2 or 4-5 of the core was sufficient to prevent the competitor from competing with the WT labeled probe.

Supplemental Table 1. Characteristics of WT and mutant UmuDAb forms

Form of UmuDAb	K_D (nm)	K_D fold increase relative to WT	Bmax*	Hill coefficient (95% CI)
WT	38.9		0.96	4.9 (3.7-7.5)
A83Y	69.2	1.8	0.99	4.7 (3.1-7.4)
HTH1	99.4	2.6	0.94	10.8 (6.4-?)
HTH2	114.2	2.9	0.78	6.1 (3.7 -?)
N100D	306.4	7.9	0.86	4.8 (3.3-6.8)
G124D	389.6	10.0	0.89	4.8 (3.7-6.3)
W192X	540.5	13.9	0.60	6.1 (4.0-10.8)
R201X	353.1	9.1	0.71	unstable

*Bmax = maximal binding as calculated by GraphPad Prism, ranging from 0-1.
? denotes inability of GraphPad Prism to determine an upper bound.
